# Characterizing and controlling infrared phonon anomaly of bilayer graphene in optical-electrical force nanoscopy

**DOI:** 10.1038/s41377-023-01320-1

**Published:** 2023-11-24

**Authors:** Junghoon Jahng, Sunho Lee, Seong-Gu Hong, Chang Jun Lee, Sergey G. Menabde, Min Seok Jang, Dong-Hyun Kim, Jangyup Son, Eun Seong Lee

**Affiliations:** 1 Hyperspectral Nano-imaging Team, Korea Research Institute of Standards and Science, Daejeon, 34113 Republic of Korea; 2 Department of Aerospace Engineering, University of Illinois at Urbana-Champaign, Urbana, IL 61801 USA; 3 Multiscale Mechanical Properties Measurement Team, Korea Research Institute of Standards and Science, Daejeon, 34113 Republic of Korea; 4 School of Mechanical Engineering, Chonnam National University, Gwangju, 61186 Republic of Korea; 5 School of Electrical Engineering, Korea Advanced Institute of Science and Technology, Daejeon, 34141 Republic of Korea; 6 Functional Composite Materials Research Center, Korea Institute of Science and Technology, Jeonbuk, 55324 Republic of Korea; 7 SKKU Advanced Institute of Nanotechnology, Sungkyunkwan University, Suwon, 16419 Republic of Korea; 8 Division of Nano & Information Technology, KIST School, University of Science and Technology, Seoul, 02792 Republic of Korea

**Keywords:** Imaging and sensing, Sub-wavelength optics, Infrared spectroscopy

## Abstract

We, for the first time, report the nanoscopic imaging study of anomalous infrared (IR) phonon enhancement of bilayer graphene, originated from the charge imbalance between the top and bottom layers, resulting in the enhancement of E_1u_ mode of bilayer graphene near 0.2 eV. We modified the multifrequency atomic force microscope platform to combine photo-induced force microscope with electrostatic/Kelvin probe force microscope constituting a novel hybrid nanoscale optical-electrical force imaging system. This enables to observe a correlation between the IR response, doping level, and topographic information of the graphene layers. Through the nanoscale spectroscopic image measurements, we demonstrate that the charge imbalance at the graphene interface can be controlled by chemical (doping effect via Redox mechanism) and mechanical (triboelectric effect by the doped cantilever) approaches. Moreover, we can also diagnosis the subsurface cracks on the stacked few-layer graphene at nanoscale, by monitoring the strain-induced IR phonon shift. Our approach provides new insights into the development of graphene-based electronic and photonic devices and their potential applications.

## Abstract (web summary)

Optical-electrical force nanoscopy. *F*_*dip*_, *F*_*es*_ and *F*_*vdW*_ are simultaneously applied and demodulated at the fundamental, second and third eigenmodes of a cantilever for PiFM, KPFM/EFM and topography images, respectively.

## Graphical abstract

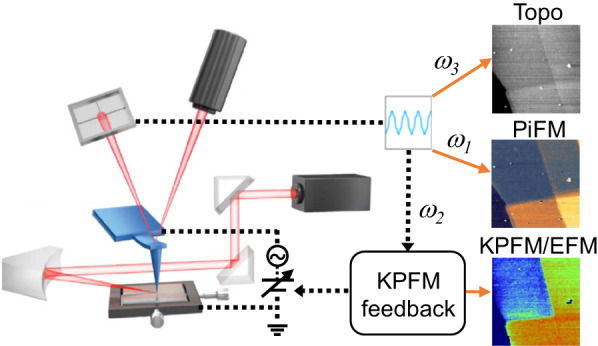

## Introduction

In recent two decades, the study of graphene has been a rapidly growing field, with the discovery of its exceptional properties and potential applications in various areas, including electronics and photonics^1–4^. Recently bilayer graphene has been focused due to its controllable band structure with respect to the gate voltage and twisted angle, providing a wider range of electronic and photonic properties. Moreover, bilayer graphene has higher mechanical strength and flexibility as well as higher thermal conductivity compared to monolayer graphene, making it suitable for designing sophisticated nanoscale devices. In particular, one such novel property comes from optical phonons which are collective vibrations of the carbon atoms that can be excited by the EM field of light.

**Fig. 1:**
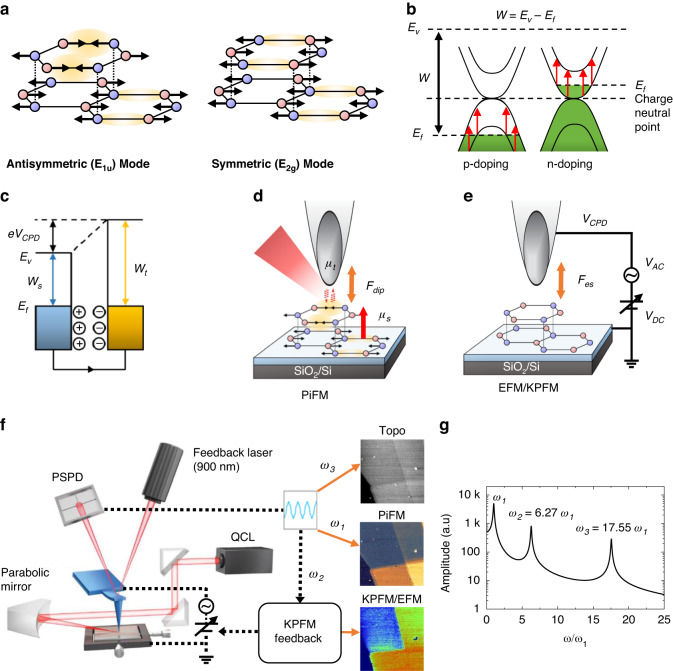
Sketch of IR phonon mode of bilayer graphene (2 L) and hybrid nanoscope of optical-electrical force detection. **a** Antisymmetric and symmetric in-plane optical phonon modes of bilayer graphene near 0.2 eV. **b** Simplified energy band diagram of bilayer graphene with respect to its doping level. The red arrow is the relevant electronic transition which contributes to the anomalous IR phonon enhancement in 2 L. **c** Diagram of contact potential difference when the two metallic materials ohmic-contact to each other. **d** PiFM and **e** EFM/KPFM configurations employing a metallic tip and bilayer graphene via *F*_*dip*_ and *F*_*es*_, respectively. **f** Sketch of hybrid nanoscope of optical-electrical force detection. *F*_*dip*_, *F*_*es*_ and *F*_*vdW*_ are simultaneously applied and demodulated at the fundamental, second and third eigenmodes of a cantilever, respectively. If the KPFM feedback is turned off, the direct electrostatic force response is measured by the EFM amplitude and phase. **g** Theoretically expected mechanical eigenmodes of a microcantilever. Typically the second and third resonances are 6.27 and 17.55 times of the fundamental eigenmode, respectively^16^

Raman scattering and infrared (IR) absorption spectroscopies have been the predominant approaches for optical phonon studies in graphene; two technologies provide complementary information due to their different selection rules^5,6^. Specifically, in bilayer graphene (2L), there exists an in-plane optical phonon modes with a frequency of approximately 0.2 eV^7–9^, known as the E_2g_ and E_1u_ modes, which are symmetric and antisymmetric modes between the layers as shown in Fig. 1a. The E_1u_ mode can only be detected through IR spectroscopy, while E_2g_ mode can only be detected through Raman spectroscopy. Note that, when we consider that graphene is a monoatomic compound, the presence of a finite IR phonon intensity in pristine undoped 2 L is quite puzzling. Because the IR active vibration requires the change of dipole moment, if all carbon atoms are equivalent, no IR intensity would be expected. This phenomenon is explained by the charged-phonon theory, which describes how the optical phonon mode borrows effective charge from the electronic transitions to which it becomes coupled^10,11^. The charged-phonon theory has been successfully applied to 2 L to explain the significant dependence of phonon intensity and Fano asymmetry on gating^12^. This effect can be utilized to determine the doping level of graphene devices since the electron doping leads to changes in the electron-phonon coupling strength^13^. In Fig. 1b, we draw the simplified band diagram of 2 L with respect to the doping levels, where the electronic transitions relevant to the anomalous IR phonon enhancement are depicted as red arrows. It can also be used to study the interaction between graphene layers and other materials, such as van der Waals materials, that can play a significant role in the performance of graphene-based devices^14,15^.

Since the IR optical phonon modes of 2 L highly depend on the spatial distribution of local doping level as well as the local strain, the comprehensive understanding between optical, electrical and mechanical information with a high spatial resolution is required to engineer the graphene-based nanoscale devices. However, the conventional optical techniques has been limited in their ability to probe individual nanostructures including the correlative information. This is because the spatial resolution of these techniques is typically on the order of a few hundred nanometers to microns, which is much larger than the size of individual nanostructures. In this purpose, the near-field imaging techniques such as tip-enhanced Raman spectroscopy (TERS) and scanning near-field optical microscopy (SNOM) are implemented to visualize the optical phonon modes in accordance with its topography at nanoscale. However, these techniques are still not able to probe the correlative optical-electrical information at nanoscale.

The local electrical property can be probed by using Kelvin probe force microscope (KPFM) with a few tens of nm spatial resolution. When in contact, two dissimilar metals or semi-conductor materials generate a contact potential difference (*V*_*CPD*_) as sketched in Fig. 1c. The V_CPD_ between the sharp metallic probe and the sample provides a means to monitor the local electrical doping level of the sample, by retrieving its work function (*W*). Here, we present a novel approach to characterize and control the IR phonon anomaly of a bilayer graphene by combining photo-induced force microscope (PiFM) with KPFM to develop the optical-electrical hybrid nanoscope through the force detection methods. By simultaneously measuring the nano-IR response and surface potential of 2 L, we figured out that there is an anomalous IR phonon enhancement of 2 L and it is originated from the charge imbalance between layers. We also demonstrate that this anomalous response can be controlled by the chemical (doping effect via Redox mechanism) and mechanical (triboelectric effect by a highly doped cantilever) approaches. Furthermore, we extend our study to more complex example such as the extrinsically stacked few-layer graphene (FLG) by diagnosing its crystallinity such as lattice mismatch or incomplete recombination, and the subsurface cracks by monitoring the strain-induced IR phonon shift. We believe that this technique has the potential to revolutionize the fields of materials science by providing new insights into the optimization of 2-D material-based electronic and photonic devices.

## Results

An optical-electrical hybrid nanoscope is developed to observe a correlation between the IR response, doping level and topographic information. We modified the amplitude modulated (AM) multifrequency atomic force microscope^16^ platform to combine photo-induced force microscope^17,18^ (Fig. 1d) with electrostatic force/Kelvin probe force microscope (EFM/KPFM)^19,20^ (Fig. 1e) through force detection method. The configuration of the hybrid nanoscope in Fig. 1f is relatively simple to the frequency modulated (FM) PiFM-KPFM system^21^ which requires the complex frequency modulation electronics such as phase lock loop (PLL) and auto gain control in the feedback loop, so that it is easy to implement in the conventional AFM system. The first three eigenmodes of a gold-coated Si tip (*PPP-NCLAu*, from Nanosensors) is plotted in Fig. 1g. In our system, the third eigenmode is allocated for the feedback control of tip-sample gap, thus provides topographic information. When the tip is driven by the mechanical piezo electric oscillator at the third eigenmode (${\omega }_{3}$) of a cantilever and engaged to the sample surface, it feels a gradient of van der Waals force (*F*_*vdW*_) to generate the gap control signal. Applied to the second eigenmode (${\omega }_{2}$) of the cantilever is the AC voltage of the frequency ω_2_ between the tip and the sample for electrical force measurements. Modulating the tip-illumination laser intensity at the sum or difference frequency between the carrier motion (${\omega }_{3}$) and the fundamental eigenmode (${\omega }_{1}$) as ${\omega }_{m}={\omega }_{3}\pm {\omega }_{1}$, the photo-induced dipole force (*F*_*dip*_) is exerted on the fundamental eigenmode of the cantilever with the heterodyne PiFM mode. Each of the three driving forces with respect to the eigenmodes can be written as below:

1 $${F}_{1}\left({{\rm{\omega }}}_{1}\right)\approx \frac{\partial {F}_{{\rm{dip}}}}{\partial z}{A}_{3}\sin \left({{\rm{\omega }}}_{1}{\rm{t}}\right)$$

2 $${F}_{2}({{\rm{\omega }}}_{2})\approx \frac{\partial C}{\partial z}\left({V}_{{\rm{DC}}}-{V}_{{\rm{CPD}}}\right){V}_{{\rm{AC}}}\sin ({{\rm{\omega }}}_{2}{\rm{t}})$$

3 $${F}_{3}\left({{\rm{\omega }}}_{3}\right)\approx {F}_{0}\sin \left({{\rm{\omega }}}_{3}{\rm{t}}\right)$$

where *F*_*0*_, *A*_*3*_, *V*_*AC*_, *V*_*CPD*_ and *V*_*DC*_, are the mechanical driving force, the amplitude of the carrier motion (third eigenmode), the AC voltage for electrostatic force between tip and sample, the contact potential difference between tip and sample, the DC voltage for KPFM feedback, respectively. The *V*_*CPD*_ is measured by turning on the KPFM feedback which nullifies the electrostatic motion at the second resonance by using the *V*_*DC*_^20^. The entire measurements in this study have been performed in the small oscillation limit so that one can regard the cantilever as the superposition of independent simple harmonic oscillators, which prevents any coupled motion between eigenmodes^22–24^. Then each force signal is demodulated at each eigenmode to obtain the amplitude and phase of each motion. The detailed calculations lie in the Section 1 of Supplementary information.

**Fig. 2:**
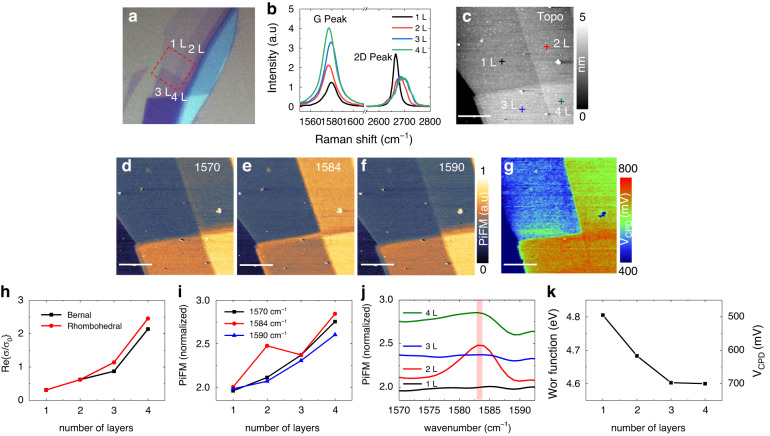
Measuring anomalous IR phonon enhancement of bilayer graphene in the exfoliated few-layer graphene on SiO_2_ substrate by using PiFM-KPFM. **a** Optical microscope image of exfoliated FLG. **b** Spontaneous Raman measurement of FLG by using far-field Raman spectroscope (Alpha 300 from *Witec*). **c** Topography, PiFM images at (**d**). 1570 cm^−1^ (off resonance), (**e**). 1584 cm^−1^ (on resonance) (**f**). 1590 cm^−1^ (off resonance) and (**g**). Contact potential difference map of the sample. The white scale bar is 4 μm. **h** Calculation of real part of relative conductivity of FLG near 0.2 eV with respect to stacking structures from previous study^26^. **i** Normalized PiFM responses with respect to the number of layers. **j** Normalized PiFM spectra on each cross in the topography. **k** Calibrated work function and *V*_*CPD*_ with respect to the number of layers. All the normalizations are conducted by the PiFM signal of substrate (SiO_2_)

The photo-induced dipole force can be related to the conductivity of graphene sheet, as *F*_*dip*_ is proportional to the real part of the electrostatic reflection factor, *β*, which is given as below^25,26^:

4 $${F}_{{\rm{dip}}}\approx -\frac{3\mathrm{Re}\left\{\beta \right\}}{4{{\rm{\pi }}{\rm{\varepsilon }}}_{0}{z}^{4}}{\rm{|}}{\alpha }_{t}{E}_{0}{{\rm{|}}}^{2}$$

with $\beta \approx \frac{\varepsilon -1+i4\pi \sigma q/\omega }{\varepsilon +1+i4\pi \sigma q/\omega }$, where *z* is the tip-sample distance, ${\alpha }_{t}$ is the polarizability of the tip, ${E}_{0}$ is the incident optical field, *ε* is relative permittivity of substrate, *σ* is conductivity of graphene, *ω* is frequency of incident light, *q* is in-plane photon momentum. The data presented in Fig. 2h is taken from the previous study^26^, which is the theoretically calculated real part of graphene’s conductivity around 0.2 eV for both Bernal and Rhombohedral structures, ranging from a monolayer (1 L) to tetralayers (4 L). It was calculated using the tight-binding Hamiltonian and Kubo formula, where only electronic contribution is considered without taking into account the effect of IR phonon modes^26,27^. Thus, under the vibrational off-resonance condition, we expect that the PiFM response is proportional to the number of layers *n* because, when the conductivity is small enough, the real part of $\beta$ in Eq. 4 is approximately proportional to the conductivity written as σ = *n*σ_0_^28^, where σ_0_ and *n* are the conductivity of 1 L and number of graphene layers, respectively. In this work, we have confirmed that it is really the case, and found that the PiFM signal around a vibrational phonon resonance shows an interesting anomalous behavior of 2 L, which is not simply proportional to the number of layers *n*.

We prepared exfoliated FLG on a SiO_2_ substrate as the optical image in Fig. 2a and conducted Raman spectroscopic measurement to determine the number of layers in Fig. 2b. The 1 L was clearly characterized by the narrow 2D mode, and the linear increase in the Raman G mode intensity indicated that the graphene consisted of 1 L to 4L^29–32^. Figure 2c presents the topography of four different graphene thicknesses taken in the red dashed rectangular area in the optical image of Fig. 2a. The PiFM measurements at off-resonance frequencies of 1570 cm^−1^ and 1590 cm^−1^ are also conducted to confirm the number of layers. As shown in Fig. 2d, f, the PiFM responses increase with the number of layers, which well corresponds to the amplitude of s-SNOM measurement in section 5 of Supplementary Information. From the fact that the amplitude of s-SNOM is directly proportional to the effective polarizability and the calculated thermal expansion of 2 L at the off-resonance is under the system noise level (sub pm/(mW/cm^2^)), it is deduced that the dominant interaction force in PiFM is the photo-induced dipole force (PiDF) rather than the photo-induced thermal force (PiTF)^33,34^. By normalizing the PiFM responses on each layer with that of the SiO_2_ substrate, a proportional increase at the off-resonance (1570 cm^−1^ and at 1590 cm^−1^) is well represented in Fig. 2i, as indicated by the black line-squares and blue line-triangles, respectively. In contrast, at the 1584 cm^−1^ IR phonon resonance, the PiFM image in Fig. 2e clearly shows that the IR response of bilayer is anomalously enhanced. The normalized line plot (red line dots) in Fig. 2i emphasizes the anomalous IR response of bilayer.

The PiFM spectra on four different layers of the sample in Fig. 2j help to comprehensively understand the anomalously enhanced IR phonon response of the 2 L. The Fano-shaped spectrum is observed like as the previous far-field measurement in doped system^8,11,12,35^. The IR signal on the bilayer at 1584 cm^−1^ is more significantly enhanced than that on the other layers. Given that the PiFM signal is proportional to the IR dipole moment, this effect may be explained by a charge imbalance between the top and bottom layer of 2 L, which originates from the charge transfer between the bottom layer and the SiO_2_ surface via Redox mechanism, resulting in the spontaneous P-doping on the bottom layer^36–38^. Since the doping effect at the graphene interface is getting screened for more than bilayer, it can be presumed that the IR response on the phonon resonance of E_1u_ mode is strongest in the bilayer. To support the suggested scenario, we measured the ${V}_{{\rm{CPD}}}$ of sample using KPFM, as shown in Fig. 2g. The surface potential (or work function) of tip is calibrated by using a known sample as ${W}_{{\rm{tip}}}={W}_{{\rm{sample}}}+e\,{V}_{{\rm{CPD}}}$, which is around 5.3 eV in section 3 of Supplementary Information. The ${V}_{{\rm{CPD}}}$ image indicates that the 1 L has a positive doping of 0.2 eV, which corresponds to the work function of 4.8 eV. In Fig. 2k, it is observed that the work function reaches the graphite value (4.6 eV) as the increase of the number of layers, which implies the spontaneous P-doping effect almost disappears after 3 L.

**Fig. 3:**
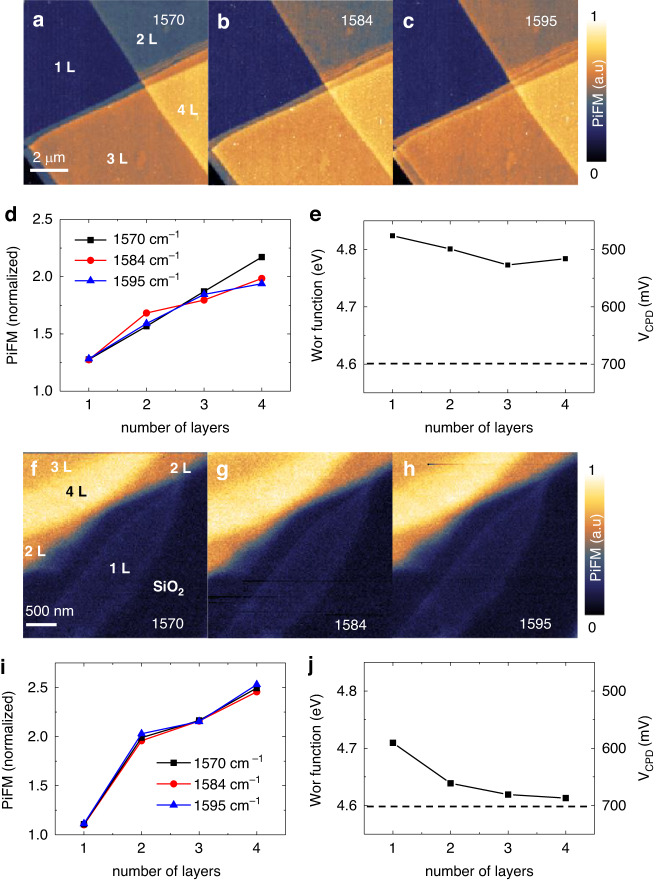
Extinguishing the anomalous IR phonon enhancement of bilayer graphene by reducing the potential difference between the layers. PiFM images after chemically P-doping the top layer by the water and ethanol at (**a**). 1570 cm^−1^ (off resonance), (**b**). 1584 cm^−1^ (on resonance) and (**c**). 1595 cm^−1^ (off resonance). The white scale bar is 2 μm. **d** Normalized PiFM signals from the data in Fig. 3a–c with respect to the number of layers. **e** *V*_*CPD*_ and calibrated work function with respect to the number of layers. PiFM images of FLG on plasma cleaned SiO_2_ substrate at (**f**). 1570 cm^−1^ (off resonance), (**g**). 1584 cm^−1^ (on resonance) and (**h**). 1595 cm^−1^ (off resonance). The white scale bar is 500 nm. **i** Normalized PiFM signals from the data in Fig. 3f–h with respect to the number of layers. **j** Calibrated work function and *V*_*CPD*_ with respect to the number of layers. All the normalizations are conducted by the PiFM signal of substrate (SiO_2_)

According to Ref. ^35^, there can be a threshold potential difference between layers to exhibit the anomalous IR phonon enhancement. Building on this concept, we endeavored to reduce the charge imbalance between layers by positively doping the top layer with ethanol/water, resulting in a comparable electrostatic potential in both layers. As plotted in Fig. 3e, the *V*_*CPD*_ between layers is reduced, and the entire work function curve shifts upward compared to the Fig. 2k, indicating that all the layers are similarly p-doped. Notably, the PiFM image at the 1584 cm^−1^ IR resonance in Fig. 3b exhibits a diminished response of anomalous phonon behavior in 2 L, with the PiFM signal being proportional to the number of layers, akin to the off-resonance images in Fig. 3a, c. The normalized PiFM curve, depicted as red line-dots in Fig. 3d, demonstrate the proportional increase with respect to the number of layers, akin to the slopes observed at 1570 cm^−1^ and 1595 cm^−1^. These findings provide compelling evidence that the anomalous IR phonon response of 2 L can be effectively controlled through chemical doping.

Furthermore, to minimize the charge imbalance between the layers, we prepared FLG on a plasma-cleaned SiO_2_ substrate, where the p-doping on the graphene-SiO_2_ interface is reduced due to the decreased water layer on the substrate. As depicted in Fig. 3j, the potential difference between layers is reduced, and the entire work function curve shifts downward compared to the Fig. 2k, suggesting that all the layers are only minimally p-doped. As expected, the anomalous phonon enhancement of 2 L is extinguished in Fig. 3g. The normalized PiFM curve shapes on the 1584 cm^−1^ IR resonance (red line-dots) in Fig. 3i closely resemble the off-resonance conditions (1570 cm^−1^ and 1595 cm^−1^), displaying a proportional increase. Based on the results in Fig. 3e, j including Fig. 2k, we can estimate that the threshold gap potential for the anomalous IR phonon enhancement of 2 L lies within the range of 100 meV to 200 meV. These findings conclusively demonstrate that the anomalously enhanced IR phonon of 2 L can be deactivated by reducing the charge imbalance between the layers.

**Fig. 4:**
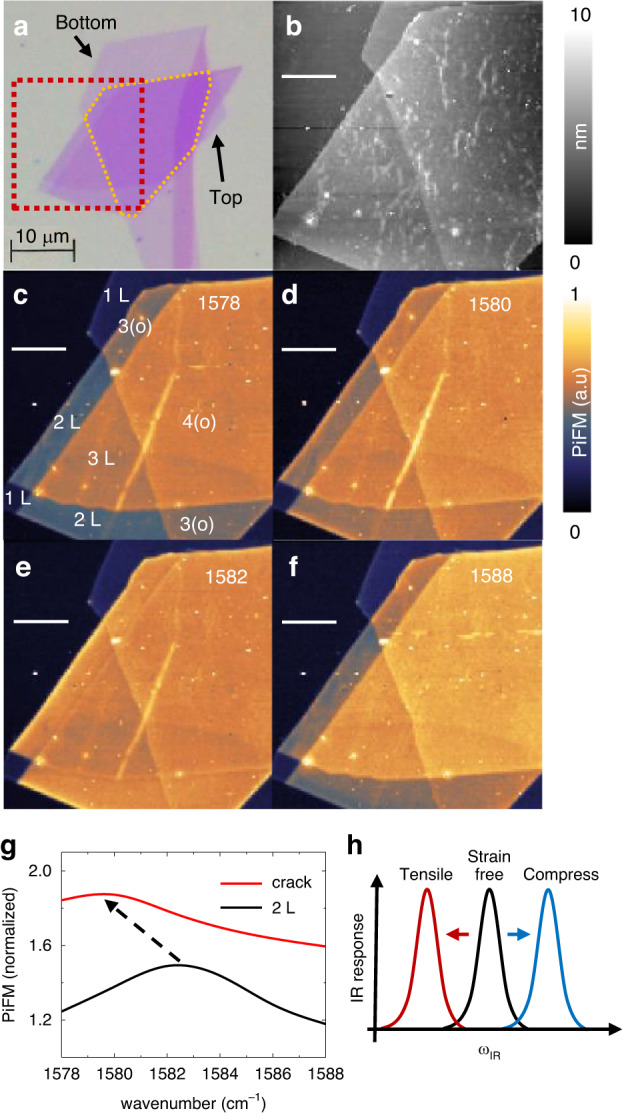
Visualizing the subsurface structure of extrinsically stacked FLG. **a** Optical image. The orange dashed line is the overlapped area. **b** Topography of the red dashed rectangular region in Fig. 4a. PiFM images of the same region at (**c**). 1578 cm^−1^, (**d**). 1580 cm^−1^, (**e**). 1582 cm^−1^ and (**f**). 1588 cm^−1^. The white scale bar is 4 μm. **g** Normalized PiFM spectral shift on the crack. **h** Sketch of infrared phonon shift of layered graphene with respect to the compress or tensile strains. All the normalizations are conducted by the PiFM signal of substrate (SiO_2_)

The in-plane optical phonon anomaly of 2 L can be influenced by the crystallinity of the sample, as well as defects and disorder. The extrinsically stacked graphene layers with the transfer method^39^, which commonly suffer from lattice mismatch or incomplete recombination, may show different IR phonon response compared to the exfoliated layers. We prepared a 1 L on a SiO_2_ substrate via exfoliation, followed by the transfer of an additional exfoliated FLG onto the 1 L, using PMMA as the transfer agent. The overlapping region is depicted by the orange dashed line in the optical image displayed in Fig. 4a. The red dashed area is analyzed using PiFM, yielding the topography shown in Fig. 4b. While this topography visualizes wiggles and contaminants such as residual PMMA present on the transferred graphene, it cannot characterize the subsurface structural information including cracks and lattice mismatches.

The PiFM images in Fig. 4c–f, on the other hand, are able to diagnose the subsurface structural information, including the crystallinity of the graphene layers. As depicted in Fig. 4c at 1578 cm^−1^, when the measurement is conducted at off-resonance, the PiFM signal is influenced only by the conductivity of graphene, enabling clear differentiation of the number of stacked layers. The trilayers in overlapping region, denoted as 3(o), exhibit incomplete lattice recombination compared to the trilayers in non-overlapping region, 3 L, by showing a slightly lower PiFM signal. At the IR phonon resonance of 1582 cm^−1^ in Fig. 4e, the PiFM response in the bilayer in non-overlapping region, 2 L, experiences significant enhancement, rendering them comparable to the responses observed in 3 L. The tri- and tetra- layers in overlapping region, 3(o) and 4(o), exhibit a similar contrast to that of 2 L and 3 L. This can be attributed to the fact that the lattice structure in the upper FLG is not strongly recombined with the bottom 1 L.

Additionally, a subsurface crack shown as a bright narrow straight line in the PiFM images (Fig. 4c–e) can be identified through the frequency shift of the IR phonon resonance, which is not featured on the topography in Fig. 4b. The bright narrow line shows maximum intensity near 1580 cm^−1^ and slightly negative contrast at 1588 cm^−1^. This can be explained by the strain-induced phonon shifts, as previously reported in the literature^40,41^. If the tensile (compress) strain is applied to the layered graphene, the IR phonon resonance redshifts (blueshifts), as sketched in Fig. 4h, respectively. By analyzing the IR-PiFM spectrum on the crack, we can directly see the redshift of the IR resonance in Fig. 4g.

**Fig. 5:**
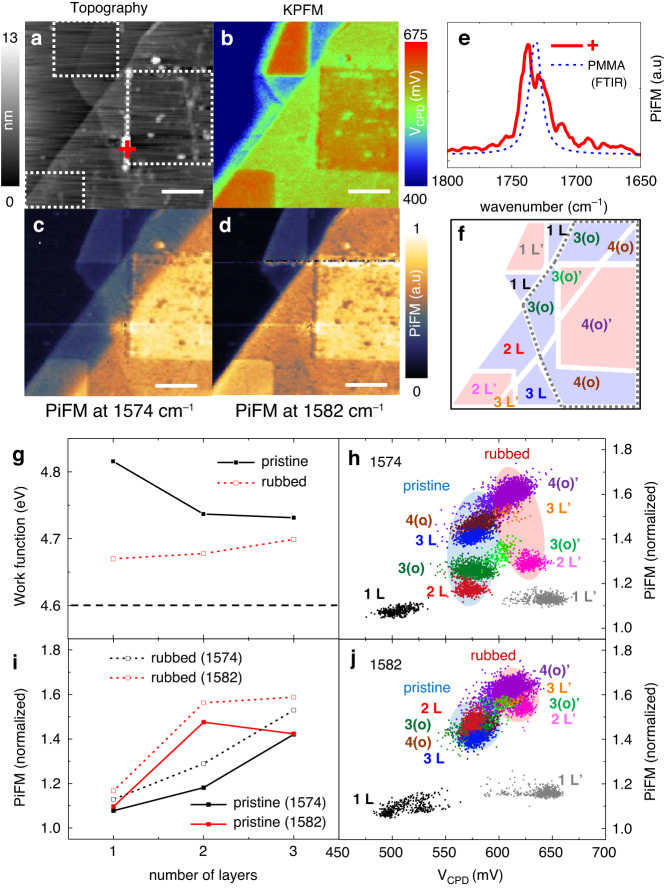
PiFM-KPFM measurements of extrinsically stacked FLG under triboelectric control. **a** Topography and (**b**). *V*_*CPD*_ map of extrinsically stacked FLG. PiFM images at (**c**). 1574 cm^−1^ (off resonance) and (**d**). 1582 cm^−1^ (on resonance). The white scale bar is 2 μm. **e** PiFM spectrum on the PMMA aggregation (red cross in the topography). **f** Block diagram of pristine regions (blue shaded) and rubbed (red shaded) regions, taking into account the pristine and overlapping area (gray dashed area), denoted as L and (o), respectively. **g** Work function and (**i**). Normalized PiFM response of pristine (solid line-squares) and rubbed (dashed line-squares) areas with respect to the number of layers. *V*_*CPD*_ vs PiFM correlation plot at (**h**). 1574 cm^−1^ (off resonance) and (**j**). 1582 cm^−1^ (on resonance). All the normalizations are conducted by the PiFM signal of substrate (SiO_2_)

Since the anomalous IR phonon of 2 L is enhanced by the charge imbalance between the top and bottom layers, this may be locally controlled by friction charging with high spatial resolution, which occurs when the p-doped graphene layer is frictionally brought into contact with the n-doped cantilever. We rubbed the extrinsically stacked graphene layers, indicated by the white dashed rectangular areas in topography of Fig. 5a, by using the highly n-doped cantilever, *PPP-NCHR* probe from *Nanosensors*, with the soft contact force of 25 nN to avoid the sample damage. The aggregation is the PMMA residue which is demonstrated by the PiFM spectrum on it in the Fig. 5e. Note that the very thin PMMA residue on the transferred FLG is not directly measured in PiFM. This is because the dominant PiFM signal on the organic polymers is based on the photoinduced thermal force which is proportional to the volume of the sample with the dissipative spectral line shape^34,24^.

In Fig. 5b–d, we present the simultaneous measurements of the surface potential and IR response of the sample. In Fig. 5b, the *V*_*CPD*_ map shows that the transferred top layer (green colored area) is already p-doped due to its contact with the PMMA surface, and its work function is approximately 4.74 eV. This value is slightly lower than the p-doped bottom 1 L on the SiO_2_ surface (light blue colored area) whose work function is around 4.81 eV. On the other hand, the *V*_*CPD*_ in the rubbed areas (red colored) are increased by around 50–150 meV, which corresponds to the decreased work function by that amount. In Fig. 5g, the line plots of work functions in the pristine (black solid lines) and rubbed (red dashed lines) areas show that the work function in the rubbed area is decreased due to this triboelectric effect. However, it is essential to note that the sample still remains positively doped since the work function (indicated by the red dashed line) is higher than the charge neutral point (also known as a *Dirac* point in a graphene) of our sample, which is around 4.6 eV (black dashed line).

The block diagram of each domain in Fig. 5f proves useful for intuitively connecting the optical, electrical, and topographical information of the intricate sample, featuring various combinations of rubbed/pristine areas with overlapping/non-overlapping graphene layers. The blue shaded regions represent the pristine areas, whereas the red shaded regions indicate the rubbed areas denoted as prime. The pentagon shaped area by gray dashed line represents the overlapping region which is denoted as (o) respectively, while the other area represents the non-overlapping region.

In Fig. 5c, captured at the 1574 cm^−1^ off resonance, the PiFM image of the rubbed areas reveals a heightened signal level compared to the pristine areas. This phenomenon arises because the PiFM signal is proportional to the photo-induced dipole, which is more sensitive to electron density than hole density. At the IR phonon resonance of 1582 cm^−1^ in Fig. 5d, despite the overall increased IR response in the rubbed areas (red shaded) of the sample, the distinctive IR phonon enhancement of 2 L’ is relatively diminished. Notably, the contrast of rubbed trilayer, 3 L’, is slightly higher than rubbed bilayer, 2 L’, while 3 L keeps lower contrast than 2 L.

The line graphs in Fig. 5i quantitatively reveal the relationship between normalized PiFM signals and triboelectric effect. At the 1574 cm^−1^ off resonance, the PiFM signals on the rubbed areas, 1 L’, 2 L’ and 3 L’ (black dashed line-squares) shifts upwards by approximately 10% from that on the pristine areas, 1 L, 2 L and 3 L (black solid line-squares), maintaining similar layer dependence. At the 1582 cm^−1^ IR resonance, the PiFM signal on pristine 2 L (red solid line-squares) surpasses that on pristine 3 L by 6%, while the signal on rubbed 2 L’ (red dashed line-squares) is 3% lower than that on rubbed 3 L’.

The PiFM-KPFM correlation graphs offer valuable insights into understanding the comprehensive interplay between the IR response and friction charging with respect to the stacking structures. The correlation graphs presented in Fig. 5h, j indicate that the impact of friction charging is more pronounced on the monolayer compared to multiple layers. Notably, the *V*_*CPD*_ experiences a 150 mV increase from 1 L to 1 L’, whereas 2 L’, 3 L’, 3(o)’, and 4(o)’ exhibit a smaller shift of approximately 50 mV from their respective pristine areas. These trends are delineated in two ellipsoidal areas of data, with the pristine layers represented by the blue shaded ellipsoidal region and the rubbed layers by the red shaded ellipsoidal region.

The data distribution observed at the 1574 cm^−1^ off resonance is compressed at 1582 cm^−1^ on the IR phonon resonance. The distinctive bilayer enhancement is perceptible exclusively in the pristine 2 L, showcasing a 2 L (red) surpass that of 3 L (blue) in PiFM responses. In contrast, the on-resonance anomalous IR enhancement in the rubbed area displays a relatively reduced effect while preserving the same layer sequence. Furthermore, given that the trilayer and tetralayer in overlapping region, 3(o) and 4(o), show akin behavior to pristine 2 L and 3 L, the PiFM signal is slightly higher in 3(o) than 4(o) on resonance, while the response in rubbed 3(o)’ is lower than rubbed 4(o)’. This PiFM-KPFM correlation graph serves as a clear representation of the intuitive relationship between the sample crystallinity and the friction charging effect, even in the complex systems.

## Discussion

We develop a novel hybrid nanoscale optical-electrical force imaging system and, for the first time, report the nanoimaging study of the IR phonon anomaly of bilayer graphene by changing its local doping level at the nanoscale. This can be explained by the charged phonon theory which suggests that the intensity of the optical phonon can be strongly enhanced when graphene is doped^10,42^. In particular, since the IR vibration requires the change of dipole moment, the IR phonon response can be further amplified under the charge imbalance between the graphene layers. Our results show that the remarkable strength of the anomalous IR enhancement in the bilayer, compared to other layers in FLG, can be attributed to the charge imbalance between the top and bottom layers in 2L, which can be controlled by chemical (doping effect via Redox mechanism) and mechanical (triboelectric effect by the doped cantilever) approaches. Furthermore, we confirm the threshold potential for triggering the anomalous IR enhancement, which is originated from the electronic band transition^11^. These information can be very useful in the development of graphene-based electronic and photonic devices such as chemical sensors to monitor the change in environmental parameters, including humidity, temperature, and gas concentration, which highly affect on the doping level of the surface of the devices^43–45^.

The optical-electrical hybrid nanoscope can be configured by the other manner, the peak-force IR (PFIR)-KPFM^46^, which measures the thermal expansion of sample near its IR absorption resonance with its surface potential. Recently, the Xiaoji group [46] successfully implemented this system to monitor the degradation of perovskite by analyzing the correlative response of charge doping/ion migration. The correlation plot between *V*_*CPD*_ and PFIR intensity well distinguishes the two different sites (pristine and degraded) of the perovskite, and shows good feasibility of the quality check for the perovskite. However, it is worth noting that this system requires the tip to contact the sample in order to measure its thermal expansion, which may have a limitation to highly dopant-sensitive sample which can be easily affected by the doping level of tip. Moreover, it is limited to access some thin inorganic samples such as few layered 2-D materials which have a very small thermal expansion. For instance, the thermal expansion of 2 L on the resonance of E_1u_ phonon mode is around sub pm/(mW/cm^2^) which closes to the noise level of PFIR. Thus, the PFIR-KPFM is expected to be very challenge to characterize the optical-electrical correlative response in 2 L. Furthermore, since the photo-induced dipole force (PiDF) depends on the near-field reflection factor (*β*) of a sample, the PiFM can reveal the subsurface structures of the devices as its refractive index contrast at even the out-of-resonance, where the thermal expansion of the 2-D materials is almost suppressed. In that case, our PiFM-KPFM system can be a good complement to this measurement, since the system can operate via photo-induced dipole force in non-contact manner.

The strong electrostatic potential can create additional tip-sample distance change, which may lead an artifact in the IR response. Since the KPFM feedback nullifies the contact potential difference, it is very useful to extract pure IR response of the conductive samples. This is very similar idea to the magnetic (or piezoelectric) force microscopy with KPFM feedback to extract its pure magnetic (or piezoelectric) responses^47–50^. Recently, the photo-induced force response of highly conductive polymer is purely extracted in the visible region by implementing the frequency modulated (FM) PiFM-KPFM system under the ultrahigh vacuum-low temperature^21^. The conductive polymer shows the electrostatic artifact in PiFM image without the KPFM feedback, while the (FM) PiFM-KPFM clearly removes the artifact and shows pure photo-induced response of the conductive polymer. We expect that the pure IR response of the chemical reactions and functional groups of the conductive polymers can be extracted by implementing our ambient IR-PiFM-KPFM technique. Moreover, since our configuration is the amplitude modulated (AM) PiFM-KPFM system which slightly modifies the conventional AFM setup, it does not require the massive vacuum system and the complex frequency modulation electronics such as phase lock loop (PLL) and auto gain control in the feedback loop. Thus, the (AM) PiFM-KPFM can be very common platform for commercialization.

In summary, we have presented a novel approach to observe a correlation between the IR response, doping level, and topographic information in FLG by developing the optical-electrical hybrid nanoscope through the force detection methods. Our results show that the spontaneous P-doping effect at the interface of graphene-SiO_2_ substrate anomalously enhances the IR response of 2 L. We demonstrate that the charge imbalance at the graphene interface can be controlled by chemical (doping effect via Redox mechanism) and mechanical (triboelectric effect by the doped cantilever) approaches. Finally, we extend our study to more complex and practical example, the extrinsically stacked FLG, by diagnosing its crystallinity such as lattice mismatch or incomplete recombination, and the subsurface cracks by monitoring the strain-induced IR phonon shift. By using the highly doped cantilever, we can control its local doping level and anomalous IR response at nanoscale, suggesting the feasibility to engineer the graphene devices. Our findings will have significant implications for a range of applications across various fields, such as graphene-based electronics, environmental monitoring including humidity and gas concentration, and material science of heterogeneous interface.

## Materials and methods

### Samples

The intrinsically stacked FLG samples are prepared by mechanically exfoliating graphite and transferring to a SiO_2_ (thickness of 285 nm)/Si substrate. Confocal Raman spectroscopic measurements (an excitation wavelength of 532 nm) are carried out on the sample to determine the number of layers (n) in section 2 in Supplementary Information. The extrinsically stacked FLG samples are prepared by exfoliating a 1 L on a SiO2 substrate, followed by the transfer of an additional exfoliated FLG onto the 1 L, using PMMA as the transfer agent^39^. One can find the optical image of the bottom and stacked top layer in the Section 4 of Supplementary Information.

### PiFM-KPFM hybrid nanoscope

The hybrid nanoscope is developed by modifying the VistaScope system from Molecular Vista in the United States. The QCL laser system from Block Engineering was used with a wavenumber resolution of 1 cm^−1^ and a tuning range of 765 to 1900 cm^−1^. The laser beam is side-illuminated to the sample with an angle of 40 degree by a parabolic mirror whose numerical aperture (NA) is around 0.4. The average illumination power was 5 mW with around 2λ μm diameter focal spot, and had a pulse duration of 40 ns. The gold-coated silicon cantilever, PPP-NCLAu from Nanosensors which is the 20 nm gold coated tip with the tip radius of 30 nm, is used for the measurements in ambient condition. The AC and DC voltages were simultaneously applied to the tip from the VistaScope controller to obtain electrostatic force response and contact potential difference. By implementing amplitude modulated (AM) multifrequency atomic force microscope technique, the photo-induced force microscope and Kelvin probe force microscope/electrostatic force microscope are combined. Typically the resonance frequency of PPP-NCLAu for the fundamental, second and third eigenmodes are around 150 kHz, 941 kHz and 2633 kHz. The cantilever responses to the photo-induced, the electrostatic and the van der Waals forces are demodulated at fundamental, second and third eigenmodes. The more details lie in the section 1 of Supplementary information.

### Supplementary information

Supplementary Information
