# Supplementary material for: Characterizing and controlling infrared phonon anomaly of bilayer graphene in optical-electrical force nanoscopy
Source: Light Sci Appl. 2023 Nov 24;12:281. doi: 10.1038/s41377-023-01320-1 (PMC10667502; doi:10.1038/s41377-023-01320-1)
Supplement: Supplementary file 1 — Supplementary Information [file 41377_2023_1320_MOESM1_ESM.pdf]

Supplementary Information for

Characterizing and Controlling Infrared Phonon Anomaly of Bilayer Graphene in Optical-Electrical Force Nanoscopy

Junghoon Jahng[1*], Sunho Lee[1,2], Seong-Gu Hong[3], Chang Jun Lee[3,4], Sergey G. Menabde[5], Min Seok Jang[5], Dong-Hyun Kim[6,7], Jangyup Son[6,8] and Eun Seong Lee[1*]

[1]Hyperspectral Nano-imaging Team, Korea Research Institute of Standards and Science, Daejeon 34113, Republic of Korea
[2]Department of Aerospace Engineering, University of Illinois at Urbana-Champaign, Urbana, Illinois 61801, United States of America
[3]Multiscale Mechanical Properties Measurement Team, Korea Research Institute of Standards and Science, Daejeon 34113, Republic of Korea
[4]School of Mechanical Engineering, Chonnam National University, Gwangju 61186, Republic of Korea
[5]School of Electrical Engineering, Korea Advanced Institute of Science and Technology, Daejeon 34141, Republic of Korea
[6]Functional Composite Materials Research Center, Korea Institute of Science and Technology, Jeonbuk 55324, Republic of Korea
[7]SKKU Advanced Institute of Nanotechnology, Sungkyunkwan University, Suwon 16419, Republic of Korea
[8]Division of Nano & Information Technology, KIST School, University of Science and Technology, Seoul 02792, Republic of Korea

*Corresponding authors. Email: phyjjh@kriss.re.kr (J. J); eslee@kriss.re.kr (E. S. L).

Table of contents

S1. Driving force for each eigenmode
S2. Raman measurement of exfoliated few-layer graphene
S3. Calibration of CPD by using Au-Si-Al standard sample.
S4. Optical image of the extrinsically stacked FLG on SiO2 substrate.
S5. s-SNOM measurement of the exfoliated FLG on SiO_2 substrate.

S1. Driving force for each eigenmode

The motion of the probe can be approximated by a point-mass model and described by the superposition of its eigenmodes in small oscillation limit. Because the higher eigenmodes contribute negligibly to the cantilever motion for our discussion here, we may assume that only the fundamental, second and third mechanical resonances of the beam system are significant. The dynamics of the cantilever in two degrees of freedom can be described by

$$m\ddot{z}_1 + b_1\dot{z}_1 + k_1 z_1 = F(t; z(t)) \qquad \text{(S1)}$$

$$m\ddot{z}_2 + b_2\dot{z}_2 + k_2 z_2 = F(t; z(t)) \qquad \text{(S2)}$$

$$mz_3 + b_3 z_3 + k_3 z_3 = F(t; z(t)) \qquad \text{(S3)}$$

where m, k_i and b_i are the mass, i-th spring constant and damping coefficient of the cantilever, respectively. $F(t; z(t))$ is the total external force including the mechanical driving force, F_0, electrostatic force, F_{es}, photo-induced force, F_{dip}, and other tip-sample interaction force, F_{ts}. Then the total force is given as below:

$$F(t; z(t)) = F_0\cos(\omega_3 t) + F_{\mathrm{es}}(z) + F_{\mathrm{dip}}(z) + F_{\mathrm{ts}}(z) \qquad \text{(S4)}$$

The other tip-sample interaction force has numerous forms for each experimental condition so that, in this study, we assume the force as the attractive van der Waals force in the non-contact region without considering the damping force for simplicity[1-2]. Then each force can be rewritten as below:

$$F_{\mathrm{dip}} = -\frac{3\mathrm{Re}\{\beta\}}{4\pi\varepsilon_0 z^4}|\alpha_t E_0|^2$$

$$F_{\mathrm{es}} = -\frac{1}{2}\frac{\partial C}{\partial z}[(V_{\mathrm{DC}} - V_{\mathrm{CPD}}) + V_{\mathrm{AC}}\sin(\omega_2 \mathrm{t})]^2$$

$$F_{\mathrm{vdW}} = -\frac{H_{\mathrm{eff}}R}{12}\frac{1}{z^2}$$

where $z > r_0$ and r_0 is the interatomic distance (~0.3 nm)[3]. H_{eff}, R, V_{AC}, V_{CPD} and V_{DC} are the effective Hamaker constant between tip and sample, the tip radius, the AC voltage, the contact potential difference (CPD) and the DC voltage between tip and sample for KPFM feedback to nullify the CPD, respectively. The cantilever dithers at the carrier frequency (ω_3) and the light is modulated at the sum or difference frequency between the fundamental and third eigenmodes $\omega_m = \omega_3 \pm \omega_1$. The sideband motion is generally induced by coupling ω_m with ω_3. Assuming that the motion is sinusoidal, the instantaneous tip-sample distance can be written as:

$$\begin{aligned} z(t) &\simeq z_c + z_1(t) + z_2(t) + z_3(t) \qquad \text{(S5)} \\ z_1(t) &\simeq A_1(z_c)\sin(\omega_1 t + \theta_1(z_c)) \\ z_2(t) &\simeq A_2(z_c)\sin(\omega_2 t + \theta_2(z_c)) \\ z_3(t) &\simeq A_3(z_c)\sin(\omega_3 t + \theta_3(z_c)) \end{aligned}$$

where z_c is the equilibrium position, z_1 is the coordinate of the fundamental eigenmode for the sideband-coupled photo-induced motion, z_2 is the coordinate of the motion due to the electrostatic force at ω_2, and z_3 is the coordinate of the carrier motion at ω_3. In the small oscillation limit, we can expand the van der Waals force and the photo-induced force with the below relation:

$$F(z) \approx F(z_c) + \frac{\partial \mathrm{F}}{\partial z}|_{z_c}(z - z_c) + \frac{1}{2}\frac{\partial^2 F}{\partial z^2}|_{z_c}(z - z_c)^2 + \cdots$$

where $z - z_c \simeq z_1 + z_2 + z_3$. Then, the total applied force to each eigenmode is given as below, respectively:

$$F(\omega_1) \approx \frac{\partial F_{\mathrm{dip}}}{\partial z} A_3 \sin(\omega_1 \mathrm{t}) + \frac{\partial F_{\mathrm{vdW}}}{\partial z} A_1 \sin(\omega_1 \mathrm{t}) \tag{S6}$$

$$F(\omega_2) \approx \frac{\partial C}{\partial z}(V_{\mathrm{DC}} - V_{\mathrm{CPD}}) V_{\mathrm{AC}} \sin(\omega_2 \mathrm{t}) + \frac{\partial F_{\mathrm{vdW}}}{\partial z} A_2 \sin(\omega_2 \mathrm{t}) \tag{S7}$$

$$F(\omega_3) \approx F_0 \sin(\omega_3 \mathrm{t}) + \frac{\partial F_{\mathrm{vdW}}}{\partial z} A_3 \sin(\omega_3 \mathrm{t}) \ . \tag{S8}$$

By substituting (S6) to (S8) into (S1) to (S3), each amplitude is derived as below simple Lorentzian form:

$$A_i(\omega_i) = \frac{F_i/k_i}{\sqrt{m^2(\omega'^2_i - \omega_i^2)^2 + (b_i\omega)^2}} \tag{S9}$$

with the driving forces of

$$F_1(\omega_1) \approx \frac{\partial F_{\mathrm{dip}}}{\partial z} A_3 \sin(\omega_1 \mathrm{t}) \tag{S10}$$

$$F_2(\omega_2) \approx \frac{\partial C}{\partial z}(V_{\mathrm{DC}} - V_{\mathrm{CPD}}) V_{\mathrm{AC}} \sin(\omega_2 \mathrm{t}) \tag{S11}$$

$$F_3(\omega_3) \approx F_0 \sin(\omega_3 \mathrm{t}) \tag{S12}$$

where $\omega'_i = \sqrt{(k_i - \frac{\partial F}{\partial z})/m}$. In general, b_i can be replaced as b'_i=b_i+Γ by considering the external damping coefficient, Γ.

S2. Raman measurement of exfoliated few-layer graphene

Confocal Raman spectroscopic measurements (an excitation wavelength of 532 nm) are carried out by Witec Micro-Raman Spectrometer Alpha 300. In Fig. S1**B**, the presence of a sharp 2D peak (represented by the black solid line) indicates the existence of a monolayer graphene. Meanwhile, in Fig. S1**C**, the increasing intensity of the G mode in a linear manner suggests that the graphene layers are stacked, with the number of layers ranging from 1L to 4L. The shift and shape of the 2D mode signal reveal that the stacked graphene layers are Bernal stacked graphene layers[4-6].

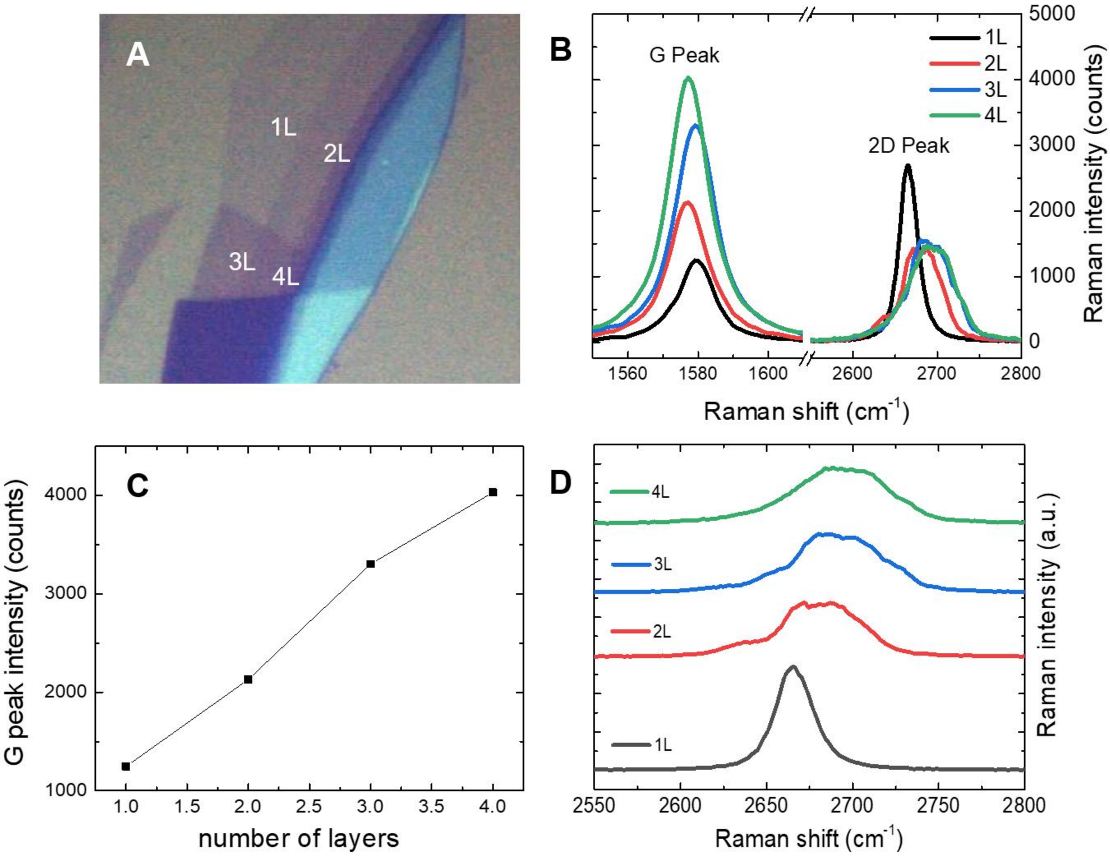

Figure S1. Raman measurements of few layer graphene. A. optical image of the FLG which is the same sample in Fig.2. **B**. Raman spectrum on each layer. **C**. Raman G peak intensity. **D**. Raman spectra of 2D mode with an offset for clarity.

S3. Calibration of CPD by using Au-Si-Al standard sample

The CPD calibration was performed using a calibration sample (PFKPFM-SMPL from *Bruker*, US), consisting of an Al-Si-Au step sample. The V_{CPD} values measured on Al, Si and Au were 1.2 V, 0.7 V and 0.1 V respectively. Considering the fact that the surface potentials of Al, Si and Au are known to be 4.1 eV, 4.6 eV and 5.1 eV respectively, by using the relation $W_{\mathrm{tip}} = W_{\mathrm{sample}} + eV_{\mathrm{CPD}}$, the work function of the gold-coated tip is estimated as 5.2 - 5.3 eV.

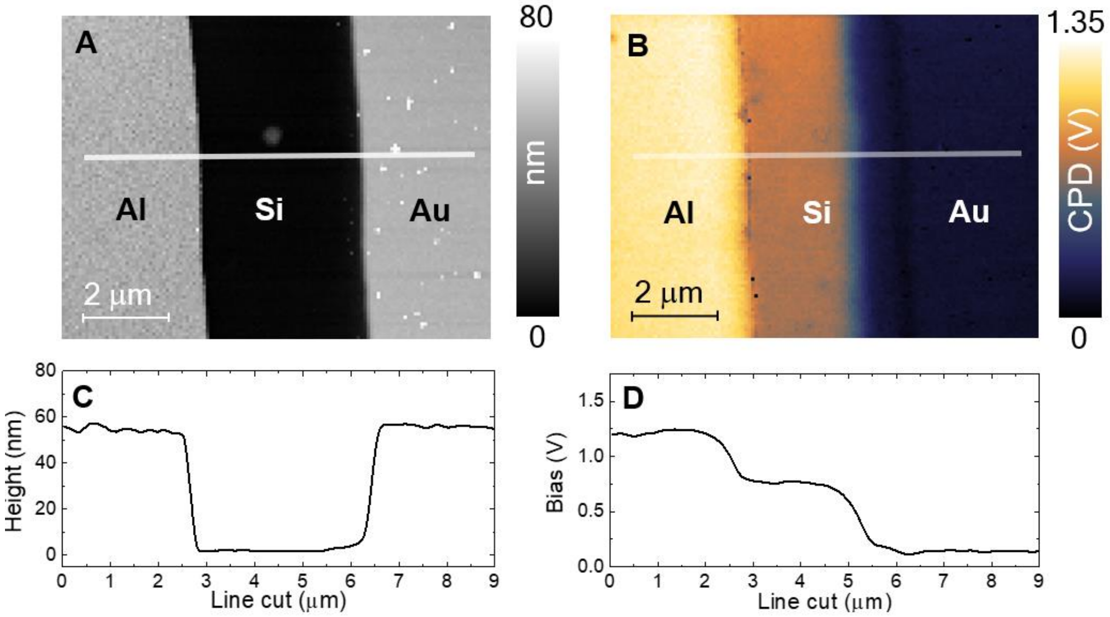

Figure S2. Calibration of CPD image by using standard KPFM sample of Al-Si-Au strip. A. Topography and **B**. CPD image of the Al-Si-Au strip. **C** and **D**. The line cuts along with the white line in the images

S4. Optical image of the extrinsically stacked FLG on SiO_2 substrate.

The extrinsically stacked FLG samples are prepared by exfoliating a monolayer graphene on a SiO_2 substrate, followed by the transfer of an additional exfoliated FLG onto the monolayer graphene, using PMMA as the transfer agent.

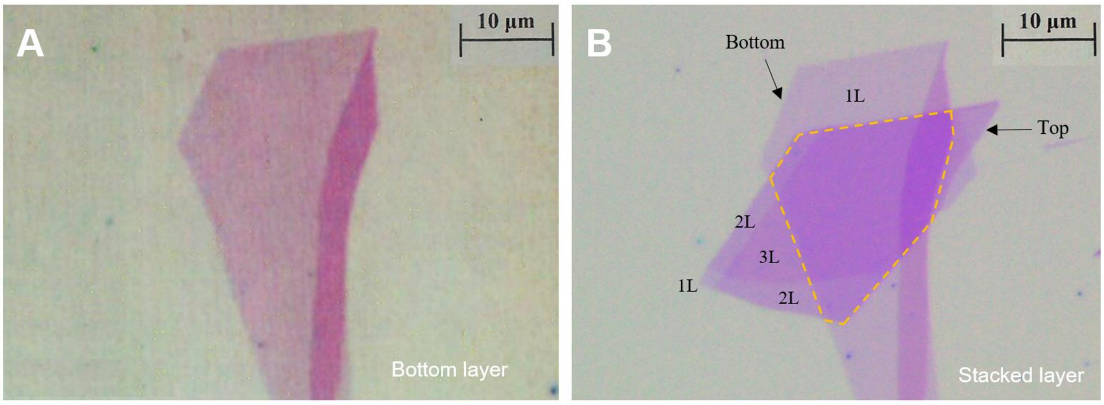

Figure S3. Optical image of the extrinsically stacked FLG on SiO_2 substrate. A. Bottom layer by exfoliating a monolayer graphene on a SiO_2 substrate. **B**. Extrinsically stacked FLG by the transfer of an additional exfoliated FLG onto the monolayer graphene, using PMMA as the transfer agent.

S5. s-SNOM measurement of the exfoliated FLG on SiO_2 substrate.

The s-SNOM measurement is helpful to characterize the origin of the photo-induced force, because the amplitude of s-SNOM is directly proportional to the effective polarizability. The s-SNOM measurement is conducted on the same sample in Fig. 2 by the neaSNOM from Neaspec GmbH coupled with the tunable QCL (MIRcat, Daylight Solutions). The Pt-coated AFM tips (ARROWNCPt, Nano World) had a typical tapping frequency Ω around 260 kHz, and the used oscillation amplitude was 60–70 nm in a non-contact mode[8]. The background-free interferometric signal[9] demodulated at the third harmonic 3Ω was used to generate all near-field images. This clearly demonstrates that the photo-induced dipole force is dominant in the few-layer graphene. This is because the thermal expansion of few layer graphene is very limited due to the graphene's high thermal conductivity.

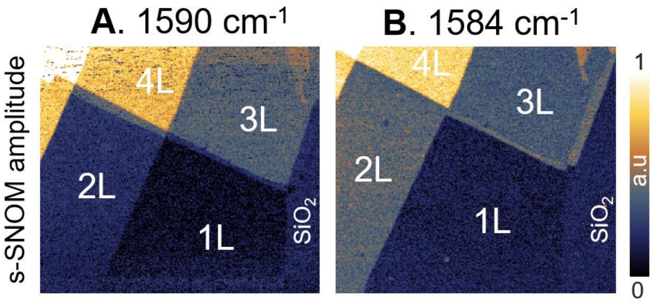

Figure S5. s-SNOM measurements of exfoliated FLG on SiO2 substrate which is the same sample in Fig. 2. s-SNOM images at **A**. 1590 cm^{-1} (off resonance) and **B**. 1584 cm^{-1} (on resonance). The image size is 16 μm x 16 μm.

References:

1. Garcıa, R.; Perez, R., Dynamic atomic force microscopy methods. *Surf. Sci. Rep.* **2002,** *47* (6-8), 197-301.
2. Jahng, J.; Kim, B.; Lee, E. S.; Potma, E. O., Quantitative analysis of sideband coupling in photoinduced force microscopy. *Phys. Rev. B* **2016,** *94* (19), 195407.
3. Garcia, R.; Herruzo, E. T., The emergence of multifrequency force microscopy. *Nat. Nanotechnol* **2012,** *7* (4), 217.
4. Nguyen, T. A.; Lee, J.-U.; Yoon, D.; Cheong, H., Excitation Energy Dependent Raman Signatures of ABA- and ABC-stacked Few-layer Graphene. *Sci. Rep* **2014,** *4* (1), 4630.
5. Hwangbo, Y.; Lee, C.-K.; Mag-Isa, A. E.; Jang, J.-W.; Lee, H.-J.; Lee, S.-B.; Kim, S.-S.; Kim, J.-H., Interlayer non-coupled optical properties for determining the number of layers in arbitrarily stacked multilayer graphenes. *Carbon* **2014,** *77*, 454-461.
6. Cong, C.; Yu, T.; Sato, K.; Shang, J.; Saito, R.; Dresselhaus, G. F.; Dresselhaus, M. S., Raman Characterization of ABA- and ABC-Stacked Trilayer Graphene. *ACS Nano* **2011,** *5* (11), 8760-8768.
7. Jahng, J.; Kim, B.; Lee, E. S., Quantitative analysis of photoinduced thermal force: Surface and volume responses. *Phys. Rev. B* **2022,** *106* (15), 155424.
8. Menabde, S. G.; Jahng, J.; Boroviks, S.; Ahn, J.; Heiden, J. T.; Hwang, D. K.; Lee, E. S.; Mortensen, N. A.; Jang, M. S., Low-Loss Anisotropic Image Polaritons in van der Waals Crystal α-MoO3. *Advanced Optical Materials* **2022,** *10* (21), 2201492.
9. Ocelic, N.; Huber, A.; Hillenbrand, R., Pseudoheterodyne detection for background-free near-field spectroscopy. *Appl. Phys. Lett.* **2006,** *89* (10), 101124.
